# HuR is exported to the cytoplasm in oral cancer cells in a different manner from that of normal cells

**DOI:** 10.1038/sj.bjc.6605084

**Published:** 2009-06-09

**Authors:** H Hasegawa, W Kakuguchi, T Kuroshima, T Kitamura, S Tanaka, Y Kitagawa, Y Totsuka, M Shindoh, F Higashino

**Affiliations:** 1 Department of Oral Pathology and Biology, Hokkaido University Graduate School of Dental Medicine, North 13 West 7, Kita-ku, Sapporo 060-8586, Japan; 2 Department of Oral and Maxillofacial Surgery, Hokkaido University Graduate School of Dental Medicine, North 13 West 7, Kita-ku, Sapporo 060-8586, Japan; 3 Department of Oral Diagnosis and Oral Medicine, Hokkaido University Graduate School of Dental Medicine, North 13 West 7, Kita-ku, Sapporo 060-8586, Japan

**Keywords:** HuR, export, AU-rich element, CRM1, oral cancer

## Abstract

HuR, a ubiquitously expressed member of the Hu protein family that binds and stabilizes an AU-rich element (ARE)-containing mRNAs, is known to shuttle between the nucleus and the cytoplasm via several export pathways. When normal cells were treated with heat shock, HuR was exported to the cytoplasm in a chromosome maintenance region 1 (CRM1)-dependent manner. However, in this study, we demonstrate that HuR is exported to the cytoplasm in oral cancer cells even if the cells were treated with the inhibitor of the CRM1-independent export pathway. Immunohistochemical and biochemical analyses showed that HuR existed in both the cytoplasm and the nucleus in oral cancer cells, such as HSC-3 and Ca9.22, but existed entirely inside the nucleus in normal cells. AU-rich element-mRNAs were also exported to the cytoplasm and stabilised in the oral cancer cells, which were inhibited by HuR knockdown. This export of HuR was not affected by at least 7 h of treatment of leptomycin B (LMB), which is an inhibitor of the CRM1-dependent export pathway. These findings suggest that HuR is exported to the cytoplasm in oral carcinoma cells in a different manner from that of normal cells, and is likely to occur through the perturbation of a normal export pathway.

HuR is a member of the ELAV (embryonic lethal abnormal vision) family of RNA-binding proteins, which has three RNA recognition motifs connected by a short hinge region (Ma *et al*, 1996). Although HuR is localised predominantly in the nucleus, it is able to shuttle between the nucleus and the cytoplasm. The export of HuR is mediated by its association with transportin 1 (Trn1) and Trn2 (Rebane *et al*, 2004) by the shuttling sequence termed ‘HNS’ in the hinge region (Fan and Steitz, 1998), and by its association with pp32 and APRIL, which includes the nuclear export signal recognised by the export receptor chromosome maintenance region 1 (CRM1) (Brennan *et al*, 2000; Gallouzi *et al*, 2001). Under physiological conditions, such as heat shock or serum stimulation, the HuR–pp32 complex is used for the transportation of AU-rich element (ARE)-containing mRNAs from the nucleus to the cytoplasm (Brennan *et al*, 2000; Gallouzi and Steitz, 2001).

An ARE is commonly present in the 3′-untranslated region of many proto-oncogenes, growth factors and cytokine mRNAs (Chen and Shyu, 1995; Jacobson and Peltz, 1996). Multiple copies of the sequence, AUUUA, often exist in the ARE and they target ARE-mRNAs for rapid degradation (Chen and Shyu, 1995; Brennan and Steitz, 2001). Numerous proteins are known to interact with AREs and modulate either the stabilisation or destabilisation of ARE-mRNAs (Chen and Shyu, 1995; Jacobson and Peltz, 1996; Antic and Keene, 1997; Brennan and Steitz, 2001). Among such proteins, HuR binds to AREs to protect ARE-mRNAs from rapid degradation (Brennan and Steitz, 2001).

In the cells transformed by an adenovirus oncogene product, ARE-mRNAs and their associated proteins, such as HuR and pp32, are exported to the cytoplasm in a CRM1-independent manner (Higashino *et al*, 2005). Therefore it is interesting to investigate the export pathway of ARE-mRNA and its binding partner proteins in non-virus-mediated tumour cells.

In this study, we provide evidence regarding the export of HuR to the cytoplasm in oral cancer cells. We found that ARE-mRNA was also simultaneously exported and accumulated in these cells and that leptomycin B (LMB) treatment failed to inhibit the HuR export. These data indicate that HuR and ARE-mRNAs are exported to the cytoplasm in oral cancer cells in a manner different from that of normal cells. In addition, these findings indicate that this HuR export can be used as a diagnostic marker for oral cancers.

## Materials and methods

### Cells and cell fractionation

Human oral cancer cells, HSC-3 (a human tongue squamous cell carcinoma cell line) and Ca9.22 (a human gingival squamous cell carcinoma cell line), and human oral normal cells, human gingival fibroblast (HGF) and periodontal ligament (PDL) cells, were cultured at 37°C with 5% CO_2_ in DMEM (Nissui Seiyaku, Tokyo, Japan) containing 10% foetal bovine serum with penicillin/streptomycin (Sigma, St Louis, MO, USA). Some of these cells were treated with heat shock (45°C, 1 h) and some were treated with LMB (Sigma) (5 ng ml^−1^) for 7 h.

Cell fractionation was carried out by separating the cells into cytoplasmic and nuclear fractions, as previously described (Weigel and Dobbelstein, 2000), by harvesting and re-suspending the cells in a fractionating buffer (10 mM Tris-HCl, pH 7.6; 150 mM NaCl; 1.5 mM MgCl_2_ and 0.5% Nonidet P-40 (Sigma-Aldrich, St Louis, MO, USA), protease inhibitor cocktail), followed by vigorous shaking for 5 min and centrifugation at 12 000 r.p.m. for 30 s. The supernatant was used as the cytoplasmic fraction. To estimate the accuracy of cell fractionation, cytoplasmic protein, *β*-tubulin (Santa Cruz Biotechnology, Santa Cruz, CA, USA), and nuclear protein, Lamin A/C (Santa Cruz), were detected by immunoblotting.

### Immunohistochemical and immunofluorescence analyses

Tissue samples were obtained from excised tongue carcinomas of patients at the Hokkaido University Dental Hospital, Hokkaido, Japan, and from normal tongue tissues. Informed consent was obtained from the patients before the samples were used. Immunohistochemical detection of HuR was carried out using the avidin–biotin complex method as previously described (Shindoh *et al*, 1996).

Cells were fixed with 4% formaldehyde, permeabilised with 0.1% Triton X-100 and incubated with antibodies specific to HuR (Santa Cruz). After incubation with HuR-specific antibodies, the cells were again incubated with FITC-conjugated secondary antibodies (Molecular Probes, Carlsbad, CA, USA). The cells were observed using an OLYMPUS IX71 (Olympus, Tokyo, Japan) fluorescence microscope.

### Western blot analysis

Western blot analysis was performed using antibodies specific to HuR (Santa Cruz), *β*-tubulin, Lamin A/C, CRM1 (BD Biosciences, San Jose, CA, USA) and *β*-actin (Sigma), as previously described (Aoyagi *et al*, 2003).

### Quantitative real-time RT–PCR

To determine the quantity of ARE-mRNAs, the cells were treated with TRI REAGENT (Sigma), after which their RNA was subjected to reverse transcription using Rever Tra Ace (TOYOBO, Osaka, Japan). For quantitative real-time RT–PCR analysis, amplification was performed in a DNA Engine-Opticon 2 PCR machine (MJ Research, Waltham, MA, USA) with SYBR Green PCR master mix (DyNAmo SYBR Green qPCR Kit, MJ Research) as previously described (Higashino *et al*, 2005).

To evaluate the half-life of *c-myc* mRNA, HSC-3 and HGF cells were treated with actinomycin D (Act.D) (Sigma) (5 *μ*g ml^−1^) for 30 or 60 min. The extracted RNA was subjected to quantitative real-time RT–PCR.

### *In situ* hybridisation

*In situ* hybridisation was performed according to a previously described method (Higashino *et al*, 2005). The cells were fixed in cold 3% formaldehyde and permeabilised with cold 0.5% Triton X-100. Hybridisation was performed overnight at 37°C in 20 *μ*l of a mixture containing 2.5 *μ*g of tRNA, 10 *μ*g of salmon sperm DNA, 2 × SSC, 0.2% BSA, 1 mM vanadyl ribonucleoside complexes, 50% formamide, 10% dextran sulphate and 10–30 ng of 3′-digoxigenin (DIG)-labelled anti-sense deoxyoligonucleotide probe (Hokkaido System Science, Sapporo, Hokkaido, Japan) for *c-fos* or *c-myc* mRNA. The coverslips were washed thrice with 2 × SSC (Invitrogen, Carlsbad, CA, USA) at 37°C and thereafter with 1 × SSC at room temperature. After washing, they were incubated for 60 min at room temperature with a dilution of 1 : 50 of anti-DIG fluorescein Fab fragments (Roche, Basel, Switzerland) in 0.2% Triton X-100/PBS containing 1% BSA (Sigma). After incubation, the coverslips were washed twice with 0.2% Triton X-100/PBS and thereafter with only PBS. The probes (sense and anti-sense) used were complementary to the nucleotides 288–328 of *c-fos* and to the nucleotides 6278–6311 of *c-myc*. Nuclei of the cells were stained using DAPI (4′,6′diamidino-2-phenylindole) (Roche).

### HuR knockdown

To knockdown HuR, siRNA was transfected using RNAiMAX (Invitrogen) as per the manufacturer's instructions. The knockdown level of HuR mRNA was analysed by western blot analysis. HuR siRNA was 5′-uuaccaguuucaauggucatt-3′, and the control siRNA was Silencer Negative Control #1 siRNA (Invitrogen).

## Results

### Localisation of HuR in oral cancer cells

**Figure 1:**
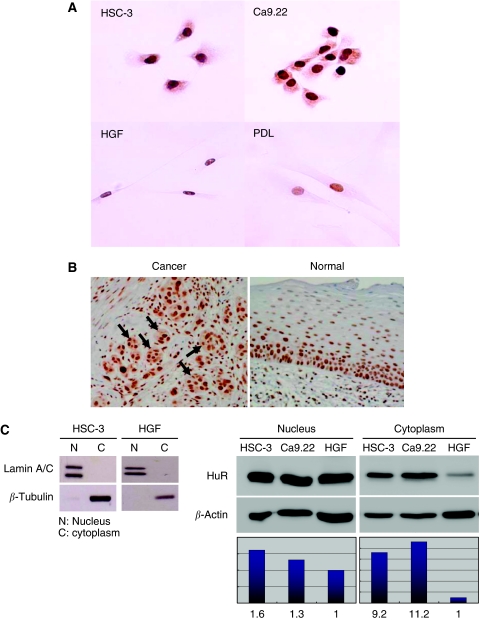
Subcellular localisation of HuR in oral cancer cells. (**A**) Immunohistochemical detection of HuR in oral cancer (HSC-3 and Ca9.22) and normal (HGF and PDL) cells. (**B**) Immunohistochemical detection of HuR in human oral cancer (tongue carcinoma, arrows) and normal oral tissues. Representative photomicrographs of HuR expression in both tissues are shown at × 200 magnification. (**C**) Western blot analysis of the nuclear and cytoplasmic fractions of HSC-3, Ca9.22 and HGF cells using HuR and *β*-actin antibodies. The fractions were analysed by antibodies against *β*-tubulin and Lamin A/C. Quantitative assessment of HuR protein in each fraction is shown as a histogram. Hold changes in the HuR level as measured by densitometry is shown.

To determine the subcellular localisation of HuR in oral cancer cells, we carried out an immunohistochemical analysis of HuR using oral cancer cells, HSC-3 (tongue carcinoma) and Ca9.22 (gingival carcinoma) cells, and normal oral cells, HGF and PDL cells. In HSC-3 and Ca9.22 cells, both the cytoplasm and the nucleus were positively stained by HuR antibody, whereas in HGF and PDL cells, only the nucleus was stained (Figure 1A). These data suggest that HuR is localised in the nucleus and cytoplasm of the oral cancer cells, whereas almost all of it is localised in the nucleus of normal cells.

Thereafter, we stained the oral cancer tissue (tongue carcinoma) with the same antibody, which showed a cytoplasmic staining of HuR (Figure 1B, arrows) as against the nuclear staining observed in the adjacent normal cells. In contrast, HuR was localised in the nucleus in normal tissues (Figure 1B). These data indicate the fact that HuR is present in the cytoplasm of oral cancer cells.

To confirm the cytoplasmic localisation of HuR, the cells were separated into cytoplasmic and nuclear fractions, and the HuR of each fraction was detected by western blotting. The amounts of HuR in the cytoplasm of HSC-3 and Ca9.22 cells were much higher than those observed in the cytoplasm of normal cells (Figure 1C). These results also suggest that HuR is localised in the cytoplasm of oral cancer cells.

Together, these results indicate that HuR is present in the nucleus of normal cells as well as in both the nucleus and the cytoplasm of oral cancer cells, which further suggest that HuR is exported to the cytoplasm in oral cancer cells.

### Export and stabilisation of ARE-mRNAs in oral cancer cells

**Figure 2:**
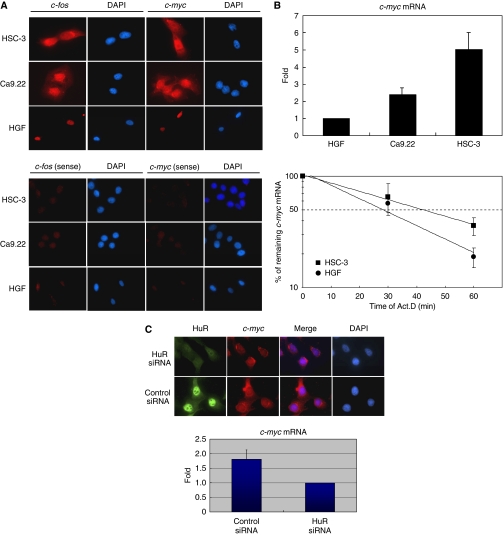
Export and stabilisation of ARE-mRNAs in oral cancer cells. (**A**) The distribution of *c-fos* and *c-myc* mRNAs in HSC-3, Ca9.22 and HGF were detected by *in situ* hybridisation using digoxigenin-labelled anti-sense (upper) and sense (lower) probes complementary to *c-fos* and *c-myc* mRNAs. The DAPI-stained nuclei are shown. (**B**) The accumulation of *c-myc* mRNAs expressed in each cell was measured by quantitative real-time RT–PCR (upper). Each cell was treated with Act.D and the amount of *c-myc* mRNA was estimated at the indicated time by quantitative real-time RT–PCR (lower). Data are mean±s.e.m. of three independent experiments. (**C**) The localisation (upper) and accumulation (lower) of *c-myc* mRNA in HuR-knockdown HSC-3 cells.

We examined the export of ARE-mRNA in oral cancer cells, as ARE-mRNA is known to be exported to the cytoplasm with its binding partner HuR (Brennan *et al*, 2000; Higashino *et al*, 2005). The subcellular localisation of *c-fos* and *c-myc* mRNAs in oral cancer cells (HSC-3 and Ca9.22) and in normal cells (HGF) was confirmed by *in situ* hybridisation. These mRNAs were detected in the nucleus and cytoplasm of HSC-3 and Ca9.22 cells, but were localised only in the nucleus of HGF cells (Figure 2A). These data suggest the export of ARE-mRNAs to the cytoplasm in oral cancer cells.

It has been previously reported that the exported ARE-mRNA is stabilised in the cells transformed with adenovirus E4orf6 (Higashino *et al*, 2005). In this study, we also examined the stabilisation of ARE-mRNA in oral cancer cells. The amount of *c-myc* mRNA expressed in oral cancer (HSC-3 and Ca9.22) and normal (HGF) cells was measured by quantitative real-time RT–PCR. Accumulation of the ARE-mRNAs was greater in the HSC-3 and Ca9.22 oral cancer cells than in the normal cells (Figure 2B). Moreover, to compare the half-life of *c-myc* mRNA, HSC-3 and HGF cells were treated with Act.D, and then the quantity of *c-myc* mRNA was measured by real-time RT–PCR. The half-life of the mRNA in HSC-3 cells was longer than that of HGF cells (Figure 2B). These results suggest the stabilisation of ARE-mRNA in oral cancer cells.

To explore the role of HuR for the export and stabilisation of ARE-mRNA in cancer cells, HSC-3 cells were subjected to HuR knockdown. In HuR-knockdown cells, *c-myc* mRNA was in the nucleus or in the perinuclear region, although the mRNA existed in both the cytoplasm and the nucleus (Figure 2C). In addition, the quantity of *c-myc* mRNA decreased in the HuR-knockdown cells compared with that in the cells transfected with the control siRNA (Figure 2C). These results indicate that the export and the increased accumulation of *c-myc* mRNA are indeed because of HuR in oral cancer cells.

### Export of HuR in the presence of LMB

**Figure 3:**
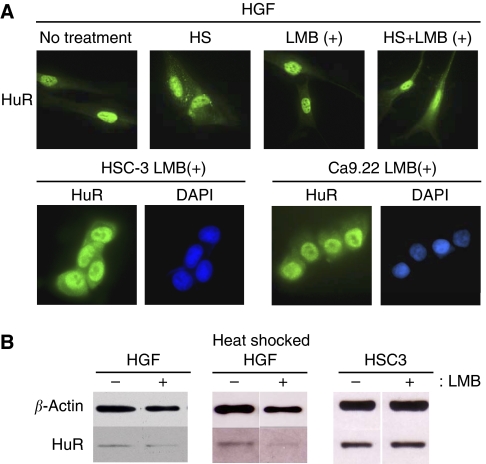
Role of CRM1 in the export of HuR in oral cancer cells. (**A**) The effect of LMB on the export of HuR was observed by an immunofluorescence analysis using HuR antibody. The HGF cells treated with heat shock in the presence and absence of LMB are shown (upper). HuR localisation in the LMB-treated HSC-3 and Ca9.22 cells, stained with DAPI are shown. (lower) (**B**) The same cells used in panel A were separated into nuclear and cytoplasmic fractions, and the amount of cytoplasmic HuR was confirmed by western blotting.

HuR is known to be exported to the cytoplasm in a manner dependent on CRM1, which is a member of the exportin family of nuclear transporters, when cells are stimulated by heat shock or serum stimulation (Brennan *et al*, 2000; Gallouzi and Steitz, 2001). On the other hand, HuR and ARE-mRNAs are known to be exported to the cytoplasm through a CRM1-independent pathway in cells transformed by an adenovirus oncogene product (Higashino *et al*, 2005). To study the export pathway of HuR in oral cancer cells, an immunofluorescence analysis of the cancer cells, to examine the localisation of HuR, was carried out after their treatment with LMB, an inhibitor of CRM1. In normal cells, heat shock treatment induced stress granules (SGs), which include HuR, and LMB treatment reduced HuR-included SGs (Figure 3A). Conversely, the accumulation of HuR to the cytoplasm in the oral cancer cells was not inhibited, even after the cells were treated with LMB for 7 h (Figure 3A).

To further confirm the effect of LMB, the existence of HuR in the cytoplasm was examined by western blotting. In normal HGF cells, HuR was observed in the cytoplasm after heat shock treatment (45°C, 1 h), but the HuR band was not visible in the lysate of the cells treated with LMB before the heat shock. On the other hand, in HSC-3 cells, HuR was observed in the cytoplasm even after the cells were treated with LMB (Figure 3B). Together, these data indicate that, in oral cancer cells, the quantity of HuR in the cytoplasms appeared to be unchanged at least 7 h treatment of LMB, and that HuR is exported to the cytoplasm by perturbing the physiological CRM-dependent export machinery.

## Discussion

This study shows that HuR and ARE-mRNAs are exported to the cytoplasm in oral cancer cells, and that ARE-mRNA accumulates in these cells. In addition, this study also shows that LMB fails to inhibit the accumulation of HuR in the cytoplasm of oral cancer cells. These results indicate that HuR and ARE-mRNAs are exported from the nucleus to the cytoplasm in oral cancer cells in a manner different from that of normal cells, and that this HuR export can be used as a diagnostic marker for oral cancer.

Cytoplasmic HuR expression has been implicated in the malignancy of several types of carcinomas, such as colon, ovary, breast, salivary, uterus, larynx and prostate cancers, and has been postulated to contribute towards the cancerous malignant phenotype (Erkinheimo *et al*, 2003; López de Silanes *et al*, 2003, 2005; Denkert *et al*, 2004; Heinonen *et al*, 2005; Cho *et al*, 2007a, 2007b; Niesporek *et al*, 2008). Our observations regarding the HuR export to the cytoplasm in oral cancer cells are similar to those previously reported in other types of carcinomas, and are thus in agreement with previous research. However, the relationship between the cytoplasmic HuR expression and the malignancy of oral carcinoma has not been established so far.

Although HuR is exported to the cytoplasm in a CRM1-independent manner in the cells transformed by an adenovirus oncogene product (Higashino *et al*, 2005), the HuR export pathway in non-virus-mediated cancer cells has never been investigated. Although the possibility that HuR had been exported and accumulated in the cytoplasm before the LMB treatment is still remaining, the inability of LMB to inhibit the HuR export in oral cancer cells, proves that the export pathway is not CRM1-dependent. The exact mechanism of HuR export in cancer cells is yet to be determined, but our results show that it occurs in a mechanism different from that of normal cells. Besides CRM1, Trn1 and Trn2 are other important nuclear export receptors that have been implicated in mediating the transport of HuR (Rebane *et al*, 2004). This gives rise to questions regarding the involvement of the Trn pathway in HuR export in oral cancer cells.

The AMP-activated protein kinase contributes towards the nuclear import of HuR (Wang *et al*, 2002), MAPK-activated protein kinase 2 increases the cytoplasmic level of HuR (Tran *et al*, 2003) and Protein kinase C facilitates the export of HuR to the cytoplasm by phosphorylation of HuR (Doller *et al*, 2007, 2008). Thus, phosphorylation of HuR is important for its localisation in the cells. Moreover, recently, HuR has been shown to be phosphorylated by Cdk1 during the G2 phase, and is retained as phosphorylated HuR in the nucleus in association with the 14-3-3 protein (Kim *et al*, 2008). Although the status of HuR phosphorylation in cancer cells has never been elucidated, HuR phosphorylation might be abnormal, which could provide valuable insights into understanding its export mechanism.
